# Iodine-Induced Hyperthyroidism—An Old Clinical Entity That Is Still Relevant to Daily ICU Practice: A Case Report

**DOI:** 10.1155/2013/792745

**Published:** 2013-04-09

**Authors:** E. Brotfain, L. Koyfman, A. Frenkel, A. Smolikov, A. Zlotnik, M. Klein

**Affiliations:** Department of Anesthesiology and Critical Care, Soroka Medical Center, Ben-Gurion University of the Negev, Beer Sheva, Israel

## Abstract

*Objective*. Hyperthyroidism has been described as elevated serum free T3 and/or free T4 levels with decreased thyrotropin (TSH) concentrations. The main causes are related to autoimmune and neoplastic pathology. However, it might be caused due to a long-term topical exposure (iodine solution dressing) or by intravenous administration of iodine-containing substances. Both clinical and laboratory features might be presented. The main management is based on interruption of all exposures with iodine solutions and also antithyroid medicine in case of severe laboratory and clinical disturbances. *Data Sources*. We present a case of iodine-induced hyperthyroidism in a critically ill ICU patient caused by excessive iodine containing antiseptic solution washes and contrast agent administration. The patient was successfully treated by discontinuing iodine exposure and beta-blocker administration. *Conclusions*. In patients with underlying thyroid gland pathology, thyroid-function tests and clinical observation in the ICU are of critical importance.

## 1. Introduction

Hyperthyroidism has been described as elevated serum-free T3 and/or free T4 levels with decreased thyrotropin (TSH) concentrations. It may present with clinical signs of heart rate and rhythm disturbances (sinus tachycardia or atrial fibrillation), muscle weakness (including respiratory muscles), weight loss, increased gut motility, ophthalmopathy and, less frequently, dermopathy [1, 2].

The most common causes of hyperthyroidism are Grave's disease (primarily in women), toxic multinodular goiter, and functional adenoma [3]. Rarely, it is induced by local inflammation of the thyroid gland (thyroiditis) or excessive intake of or exposure to iodine [4, 5]. Excessive iodine intake might be due to a long-term topical exposure (iodine solution dressing) or by intravenous administration of iodine-containing substances. In both cases the laboratory features and clinical significance are similar [6]. 

In this paper we present a case of iodine-induced hyperthyroidism in a critically ill ICU patient caused by excessive iodine containing antiseptic solution washes and contrast agent administration.

## 2. Case Report

**Figure 1:**
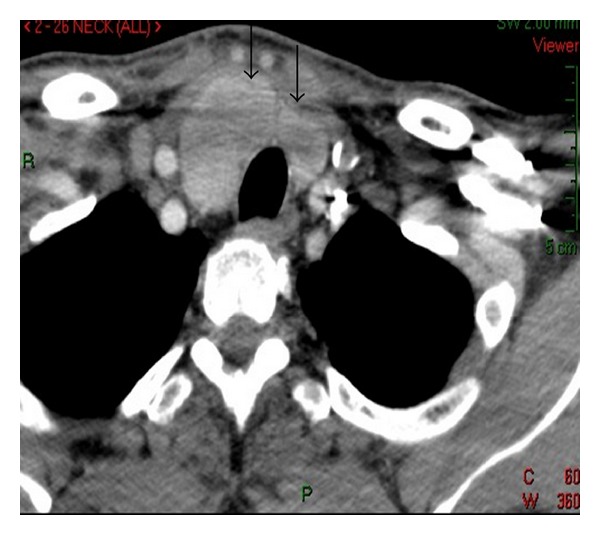
Patient's CT imaging showing a remarkable inflammatory process on the posterior portion of the neck and upper back. Note the accidental findings of diffusive enlargement of right lobe and isthmus of thyroid gland (see two black arrows) related to unrecognized subclinical goiter.

A 52-year-old man was admitted to the hospital because of sepsis from a posterior neck abscess. His past medical history included heavy smoking, dyslipidemia, diabetes mellitus type II, and coronary artery bypass graft surgery three years earlier. There was no personal or family history of thyroid disease. Ten days before admission, a furuncle developed on his posterior neck but was not treated. His baseline parameters at admission included a fever of 39.2°C and a WBC of 17,000. There was no evidence of organ failure. A CT scan of his neck with an iodine-containing contrast agent showed an extensive inflammatory process involving the subcutaneous and muscle tissue of the posterior neck, which extended to the paravertebral space at the level of C4-C5, without direct contact with the thyroid gland. The CT also showed a slightly enlarged right lobe and isthmus of the thyroid gland as an incidental finding (Figure 1). 

Treatment was initiated with broad-spectrum antibiotics (ciprofloxacin, clindamycin, and penicillin), and the patient was taken urgently to the operating room for wide incision, iodine irrigation, and abscess drainage.

In spite of antibiotic therapy, recurrent iodine irrigations, and regular changes of dressings the patient showed no clinical improvement. Within a few days he developed severe sepsis with acute respiratory failure, necessitating intubation and mechanical ventilation. At that point the patient was transferred to the intensive care unit (ICU). 

**Figure 2:**
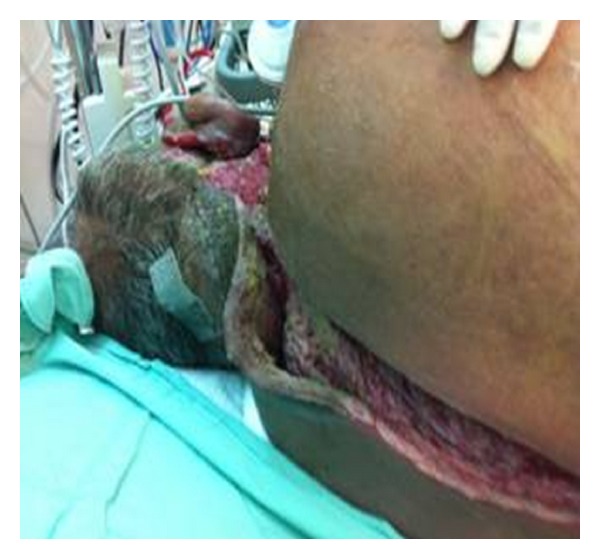
Enlarged area of necrotizing fasciitis in the posterior portion of the patient's neck and back. The area was exposed for long time to iodine-containing solutions.

Treatment in the ICU included mechanical ventilation support (at first oral tube and later via tracheostomy), antibiotics as indicated by bacteriological cultures, and extensive surgical debridement of necrotic subcutaneous tissue and muscles through the upper portion of the back (Figure 2).

**Figure 3:**
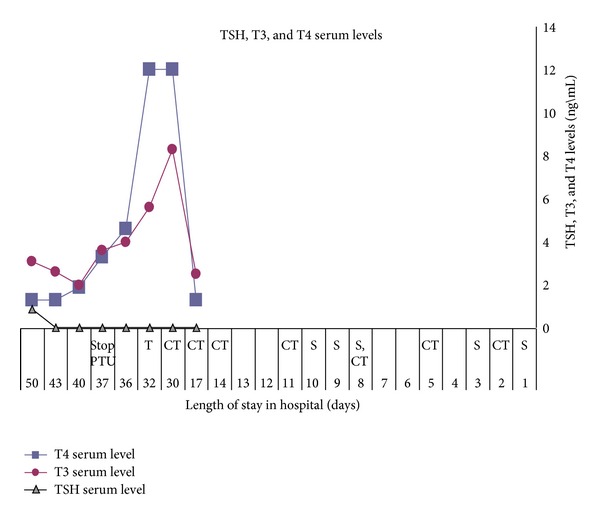
TSH, T4, and T3 serum levels during the patient's hospitalization stay. The timing of the CT scans, surgery, and treatment ( S—surgery, CT—CT scan, and T—treatment) is shown. It should be noted that all procedures were conducted with iodine-containing solutions.

At each operation the wound field was washed multiple times with iodine-containing solutions. The patient underwent two additional CT scans of the head, neck, and chest with contrast iodine to guide surgical exploration (see Figure 3 for details). 

The surgical wound started to heal with a decreasing need for debridement. The patient was weaned successfully from ventilatory support but was tachycardic, and his fever persisted as evidence of “uncontrolled sepsis.”

A review of the patient's laboratory results showed normal T4 (1.3 ng/dL), T3 (2.5 pg/mL), and low TSH (<0.05 uIU/mL) levels 10 days after admission. 

Thyroid-function testing was repeated two weeks later. The serum TSH level was still very low (<0.05 uIU/mL), while the serum T3 (8.3 pg/mL), T4 (>12.0 ng/dL), and thyroglobulin (2612.0 ng/mL; normal range <0.3–47.99 ng/mL) levels were significantly elevated. The physical examination at the time was unremarkable.

Normal sinus tachycardia, without other abnormalities, was observed on the electrocardiogram. In view of significant laboratory findings that were consistent with thyrotoxicosis, treatment was initiated with steroids and PTU (propylthiouracil). Propranolol was prescribed to control sinus tachycardia. All iodine exposure was stopped immediately and subsequently averted. The antiseptic solution used for washing and dressing the wound was changed to sulfamylon. 

Laboratory tests conducted one week later showed significant improvement in serum T3 levels to the normal range (from 8.3 to 2.6 pg/mL), and serum T4 levels (from >12 to 1.3 ng/dL). However, the serum TSH level was still at the very low range (<0.05 uIU/mL). The patient's heart rate decreased to normal and the fever diminished. In view of the quick decrease in the serum T4 level PTU therapy was discontinued (see Figure 3). The patient was returned to the ENT department and two weeks later plastic surgery for wound closure was performed successfully.

## 3. Discussion

Iodine-induced hyperthyroidism has been described previously in the medical literature. Exposure to excess iodine may come from several sources including enteral (dietary supplements and iodine-containing medications), topical (iodine antiseptic solutions), and intravenous (contrast radiographic agents) [7–9]. Normally, the thyroid gland can regulate thyroid hormone synthesis and secretion in the presence of excessive amounts of iodine in the body. This has been well described as the Wolff-Chaikoff effect or phenomenon [10, 11], based on a decrease in the expression of the sodium-iodide symporter, leading to a decrease in the transport of iodide into the thyroid gland and restoration of normal thyroid function.

However, in the case of underlying thyroid gland disease such as multinodular goiter, excess iodine exposure may cause hyperproduction of thyroid hormones and clinically significant thyrotoxicosis. This development is consistent with the present case.

As demonstrated on CT imaging, our critically ill patient had an underlying multinodular thyroid gland. Those findings were not associated with the inflammatory process itself. Moreover, the necrotizing process involved only the posterior portion of the patient's neck and back. 

The main source of iodine excess in our patient came from exposure to a significant amount of an iodine-containing antiseptic solution for a long period of time (over two weeks from admission to the hospital).

This exposure may have been complicated further by contact with the large operating area, which could have amplified the systemic absorption of iodine and the cumulative effect of iodine exposure in both the operating room and the ICU. Some authors [8, 9, 12, 13] have provided evidence for significant systemic absorption of iodine through mucosal surfaces or skin wounds. 

Moreover, in our patient there was additional potential excessive iodine exposure because he underwent multiple radiographic studies with iodine contrast agents. Martin et al. [14] published a clinical followup of seven patients who developed hyperthyroidism after contrast media radiography.

Management of iodine-induced hyperthyroidism consists primarily of discontinuation of the source of excess iodine. In our case this was not done until iodine exposure had continued for about two weeks.

The sinus tachycardia seen in our patient could be explained by other clinical causes such as sepsis, agitation, and pain. It was treated initially with the beta-blocker, propranolol at a dose of 20 mg t.i.d.

Most cases of iodine-induced hyperthyroidism are self-limited and resolve successfully a few months after cessation of exposure. However, in some cases of severe and refractory hyperthyroidism, antithyroid drug treatment has been required, usually with high doses of methimazole or PTU (propylthiouracil) [14]. In the present case we initiated treatment with PTU at a dose of 100 mg t.i.d. and hydrocortisone at a dose of 100 mg t.i.d. This therapy was discontinued after one week in light of the rapid T4, T3, and TSH laboratory response. 

## 4. Conclusion

Undiscovered and untreated hyperthyroidism is a dangerous clinical condition. It may lead to multiple organ involvement and even a life-threatening thyrotoxic storm. Because hyperthyroidism caused by ongoing exposure to iodine-containing solutions can be clinically silent there may be a delay in the initiation of treatment. A high index of suspicion for hyperthyroidism should be maintained in cases of significant, long-term exposure to iodine-containing solutions in the ICU. In patients with underlying thyroid gland pathology thyroid-function tests and clinical observation in the ICU are of critical importance.
